# Internet Tool to Support Self-Assessment and Self-Swabbing of Sore Throat: Development and Feasibility Study

**DOI:** 10.2196/39791

**Published:** 2023-12-08

**Authors:** Mark Lown, Kirsten A Smith, Ingrid Muller, Catherine Woods, Emma Maund, Kirsty Rogers, Taeko Becque, Gail Hayward, Michael Moore, Paul Little, Margaret Glogowska, Alastair Hay, Beth Stuart, Efi Mantzourani, Christopher R Wilcox, Natalie Thompson, Nick A Francis

**Affiliations:** 1 School of Computing University of Portsmouth Portsmouth United Kingdom; 2 School of Healthcare Enterprise and Innovation University of Southampton Southampton United Kingdom; 3 Local Clinical Research Network Wessex Southampton United Kingdom; 4 Nuffield Department of Primary Health Care Sciences University of Oxford Oxford United Kingdom; 5 Centre for Academic Primary Care Bristol Medical School University of Bristol Bristol United Kingdom; 6 Pragmatic Clinical Trials Unit Queen Mary University of London London United Kingdom; 7 Cardiff School of Pharmacy Cardiff University Cardiff United Kingdom

**Keywords:** sore throat, ear, neck, throat, pharyngitis, self-assessment, self-swabbing, primary care, throat, development, feasibility, web-based tool, tool, antibiotics, develop, self-assess, symptoms, diagnostic testing, acceptability, adult, children, social media, saliva, swab, inflammation, samples, support, clinical, antibiotic, web-based support tool, think-aloud, neck, tonsil, tongue, teeth, dental, dentist, tooth, laboratory, lab, oral, oral health, mouth, mobile phone

## Abstract

**Background:**

Sore throat is a common problem and a common reason for the overuse of antibiotics. A web-based tool that helps people assess their sore throat, through the use of clinical prediction rules, taking throat swabs or saliva samples, and taking throat photographs, has the potential to improve self-management and help identify those who are the most and least likely to benefit from antibiotics.

**Objective:**

We aimed to develop a web-based tool to help patients and parents or carers self-assess sore throat symptoms and take throat photographs, swabs, and saliva samples for diagnostic testing. We then explored the acceptability and feasibility of using the tool in adults and children with sore throats.

**Methods:**

We used the Person-Based Approach to develop a web-based tool and then recruited adults and children with sore throats who participated in this study by attending general practices or through social media advertising. Participants self-assessed the presence of FeverPAIN and Centor score criteria and attempted to photograph their throat and take throat swabs and saliva tests. Study processes were observed via video call, and participants were interviewed about their views on using the web-based tool. Self-assessed throat inflammation and pus were compared to clinician evaluation of patients’ throat photographs.

**Results:**

A total of 45 participants (33 adults and 12 children) were recruited. Of these, 35 (78%) and 32 (71%) participants completed all scoring elements for FeverPAIN and Centor scores, respectively, and most (30/45, 67%) of them reported finding self-assessment relatively easy. No valid response was provided for swollen lymph nodes, throat inflammation, and pus on the throat by 11 (24%), 9 (20%), and 13 (29%) participants respectively. A total of 18 (40%) participants provided a throat photograph of adequate quality for clinical assessment. Patient assessment of inflammation had a sensitivity of 100% (3/3) and specificity of 47% (7/15) compared with the clinician-assessed photographs. For pus on the throat, the sensitivity was 100% (3/3) and the specificity was 71% (10/14). A total of 89% (40/45), 93% (42/45), 89% (40/45), and 80% (30/45) of participants provided analyzable bacterial swabs, viral swabs, saliva sponges, and saliva drool samples, respectively. Participants were generally happy and confident in providing samples, with saliva samples rated as slightly more acceptable than swab samples.

**Conclusions:**

Most adult and parent participants were able to use a web-based intervention to assess the clinical features of throat infections and generate scores using clinical prediction rules. However, some had difficulties assessing clinical signs, such as lymph nodes, throat pus, and inflammation, and scores were assessed as sensitive but not specific. Many participants had problems taking photographs of adequate quality, but most were able to take throat swabs and saliva samples.

## Introduction

Sore throat is a common reason for patients to present in primary care and is thought to result in more inappropriate antibiotic prescriptions than any other condition [1]. An average-sized UK general practice, with a practice population size of 7000, has an estimated 548 consultations per year for sore throat, and over half of these will result in an antibiotic prescription [2,3]. Antimicrobial resistance is a global public health challenge, which has been accelerated by the overuse of antibiotics and is the cause of severe infections, complications, longer hospital stays, and increased mortality [4]. Unnecessary prescribing of antibiotics is also associated with an increased risk of adverse effects, more frequent reattendance in primary care and increased medicalization of self-limiting conditions [4].

Most sore throats are caused by viral infections and 90% of patients are symptom free by 1 week, whether they are treated with antibiotics or not [5]. Group A β-hemolytic streptococcus (GABHS) is the most common bacterial pathogen in sore throat, isolated in approximately 20% of the cases [6]. Complications of sore throat (including quinsy or peritonsillar abscess, acute otitis media, and acute sinusitis) caused by a GABHS infection are rare in adults and children [7].

Distinguishing between viral and bacterial infections is difficult because the signs and symptoms are similar regardless of the cause, which can result in unnecessary antibiotic prescriptions [8]. Clinical scoring systems have been developed to help clinicians identify people with acute sore throat who are more likely to benefit from antibiotics, including FeverPAIN (developed in a UK primary care setting in 2013) and Centor criteria (developed in the United States in an emergency department setting) [7]. Both scores have been shown to have moderately good diagnostic properties for identifying streptococcal sore throat in general practice (positive predictive value [PPV] 37%-47% and negative predictive value [NPV] 72%-90%) [9]. A FeverPAIN score of 4 or 5 was shown to be associated with a 62% to 65% probability of having a bacterial infection [7].

Another approach to improving the targeting of antibiotic use in patients with sore throat, which can be used alone or in combination with clinical scoring systems, is collecting throat swab samples for testing. Samples can be sent to a laboratory for culture-based testing; tested using a rapid antigen detection test, which has a sensitivity of 70%-90% for GABHS [2] and can provide a result in a few minutes; or could potentially be used for novel tests involving molecular techniques [10] and testing for evidence of host response. However, throat swabbing has traditionally only been available as part of a face-to-face consultation, and it is unclear whether patients or parents are able to self-sample at home.

The COVID-19 pandemic led to the rapid adoption of remote consulting in many countries, with about 90% of UK primary care consultations being done remotely by April 2020 [11]. There are concerns that remote consulting may lead to increases in antibiotic prescribing [12], and there will likely be increased levels of remote consulting after the pandemic compared with before COVID-19 [13]. Furthermore, early in the pandemic, guidance was issued recommending that the oropharynx should not be examined unless absolutely necessary and taking a throat swab was identified as a high-risk procedure for transmission of COVID-19 infection. The Royal College of Paediatrics and Child Health (RCPCH) also suggested adopting a pragmatic approach to assessing sore throat by automatically starting with a FeverPAIN score of 2 in lieu of an examination, which in turn may artificially inflate scores and lead to higher prescribing [14].

Supporting remote and self-management are key priorities for the National Health Service (NHS) [15]. A significant proportion of patients with sore throats could potentially be managed without the need for a health care consultation, or at least without the need for a face-to-face consultation. To facilitate safe remote management of patients with sore throats, it is important to determine how well patients and parents or carers of children can assess their own sore throats. Most of the criteria included in the FeverPAIN and Centor scores should be relatively easy for patients or parents to assess (history of fever, recent onset, no cough, or coryza). It may be possible for patients to assess tender anterior cervical nodes, pus, and inflammation on examination of the throat, but this has not yet been evaluated.

There is, therefore, a need to develop a tool to help patients self-evaluate sore throat and obtain self-collected throat swabs and saliva samples and to assess the feasibility, validity, and effects of the use of this tool in adults and children with a sore throat. However, before we can conduct an adequately powered study, we need to identify the barriers and facilitators to undertaking research in this setting and assess the feasibility of conducting a larger study. The overall aims of the Scores and Swabs to Self-Assess Sore Throat (4S) study were therefore to develop and evaluate the feasibility of using a web-based tool for patients and parents or carers to self-assess sore throat symptoms and obtain self-collected throat swabs and saliva samples. This will allow us to develop a future adequately powered study to evaluate the effects of using this tool to guide patients in deciding whether to consult with a health care practitioner and potentially guide health care practitioners on whether to prescribe antibiotics.

## Methods

### Stage 1—Developing a Web-Based Tool to Support Self-Assessment and Sampling

The web-based tool was developed following key principles of the Person-Based Approach (PBA) [16] to intervention development to ensure the tool was accessible, feasible, and optimally engaging to a wide range of target users. The first 2 stages of the PBA are planning and design. Our approach to this involved conducting a brief scoping review of scientific literature and patient materials on self-assessment of sore throat, throat swabbing, and saliva testing and then selecting the most useful aspects in consultation with 3 public contributors with experience of sore throat at weekly meetings from October 2020 to January 2021. This led to the development of a web-based support tool (which can be adapted to be paper based) to support self-assessment of sore throat with clinical scores and tests using the Lifeguide (University of Southampton) platform. Lifeguide is a set of open-source software tools that allows intervention designers with no experience of programming to create web-based interventions to support healthy behavior. The next stage of PBA is the “development and evaluation of acceptability and feasibility” in order to iteratively refine the intervention. A key component of this involves conducting real-time user studies to understand the views of the users. To do this, we set out to recruit 10-20 healthy adults and parents or carers of children (using social media and snowball sampling) to iteratively optimize the tool to support self-assessment and testing. We elicited and observed reactions to every element of the intervention, using qualitative think-aloud techniques during a video call [17], and used the PBA table of changes tool to efficiently extract the verbatim positive and negative user comments about the prototype intervention. This facilitated informed discussions about how best to refine the prototype, providing a clear record of agreed changes, their rationale, and priority [18]. At the end of this stage, we had a web-based tool that could be used to help with self-assessment of sore throat (and taking throat swabs and saliva samples).

### Stage 2—Feasibility Study

#### Aims

The aims of the feasibility study were to (1) explore participant or parent or carer views about assessment of sore throat and self-collection of throat swab and saliva samples using our web-based tool; (2) understand the feasibility of and barriers and facilitators to conducting a study to evaluate the diagnostic properties of home assessment of sore throat using clinical prediction rules and self-collected throat or saliva samples; and (3) assess the feasibility of participants using their smartphone to photograph their throat.

#### Participants

We recruited adults with acute sore throat and parents or carers of children with acute sore throat from participation in general practices and by directly using social media and adverts. The inclusion criteria were (1) ongoing sore throat for a duration of 14 days or of less, (2) able to communicate in English by videoconferencing, (3) aged 17 years or older (adult) or 3-16 years with a parent willing to consent, and (4) able and willing to comply with the study protocol. A symptom duration of 14 days was seen as a reasonable balance between feasibility (it is much more difficult to identify people within the first couple of days of symptom onset) and still being able to remember their experience. Exclusion criteria were (1) any significant disease, disorder, or finding that may significantly increase the risk to the participant, affect their ability to participate, or impair interpretation of the study data and (2) current or recent (within 3 months) involvement in a clinical trial. Participants provided consent remotely, with parents consenting on behalf of children.

#### Data Collection

Participants recorded demographic data (including age, gender, and race and ethnicity) and assessed and recorded the clinical features of their illness. This included self-assessing each of the clinical criteria from both the FeverPAIN (0-5) and Centor scores (0-4) and rating the severity of their illness (on a scale of 0-10). They also recorded whether they had been prescribed antibiotics for their sore throat and took a photograph of their throat and sent it to the study team. Participants were then sent a test kit including 2 swabs (bacterial and viral) and 2 saliva collection kits by next day delivery. Following the receipt of the test kit, participants had a video call during which they were observed (without intervention) performing the swab and saliva tests. Participants packed and sent the biological samples directly to the laboratory using prepaid postal boxes. Following the self-swabbing, participants were asked a series of questions about their experience of using the site and taking the samples in a structured interview via the video call (see Multimedia Appendix 1 for topic guide).

#### Sample Size

We aimed to recruit 40-60 participants (20-40 adults and 10-20 children) with sore throat. A total of 40 participants would allow us to provide a 95% CI of +11% or –11% around a descriptive statistic of 50%. Proportions greater or less than 50% would have greater precision.

#### Quantitative Analysis

Data completeness, including the proportion of participants able to assess each feature, was described using standard descriptive statistics. Self-assessed FeverPAIN and Centor scores and the proportion of participants able to take throat photographs are described. All photographs were assessed for quality (ability to see the posterior pharynx clearly and to assess for inflammation and the presence of pus) and, if the quality was acceptable, were then rated on the degree of inflammation (none, mild, moderate, and severe) and the presence of pus or exudate on the tonsils or posterior pharynx (non, mild, moderate, or severe) by 3 GPs independently. Interrater reliability for the clinician’s assessment of inflammation and pus was calculated. When there was disagreement among the clinical raters regarding participants with photographs of adequate quality, a final decision was made through discussion between all 3 clinical raters. Patient self-assessments were then compared to the clinician-agreed assessments to calculate the accuracy, sensitivity, specificity, PPV, and NPV of patient self-assessment. In the assessment of the feasibility and acceptability of self-sampling, we split the cohort into those aged 5 years or younger and those aged 6 years or older, as we thought that the challenges involved in obtaining samples in those aged 5 years or younger were likely to be different from older children and adults.

#### Qualitative Interviews

All stage 2 participants were invited to take part in a short 10- to 15-minute interview to discuss their experiences of self-assessing and self-testing a sore throat at home. In terms of self-assessment of their sore throat, participants were asked questions about the use of the website, their experiences of assessing a sore throat using diagnostic indicators, and their experiences of taking a photograph of the throat.

Stage 2 interviews were transcribed verbatim and analyzed using the stages of thematic analysis [19]. The process of analysis involved familiarization with the transcripts, followed by line-by-line coding to inductively generate an initial coding framework. This framework was discussed and refined in meetings with core members of the research team with qualitative expertise. The framework was organized topically, around barriers and facilitators related to the participants using the intervention to conduct self-assessments at home. Analyses were facilitated by the software program NVivo (version 12; Lumivero).

### Ethical Considerations

Ethics approval was provided by South West—Cornwall and Plymouth Research Ethics Committee (20/SW/0175). All adult participants provided written informed consent, and for all child participants, written consent was obtained from a parent or legal guardian. The data have been anonymized. Participants received no financial compensation for taking part in this study.

## Results

### Stage 1—Intervention Development

#### Participants

A total of 7 adults and 4 parent-child dyads (3 children aged 6-15 years and 1 child aged between 3 and 5 years) participated in stage 1 (intervention development).

#### Findings

##### Overview

The intervention was refined through several iterations during the stage 1 interviews. Key changes to the intervention materials resulting from user feedback related to ease of website use, clarity of instructions, and suitability of materials.

##### Ease of Website Use

The information architecture required adjustment to make it easier for users to find instructions—for example, the instructions for test kits were initially grouped by type, such that both bacterial and viral swab collection followed the same set of instructions. This was confusing to participants, so the instructions were split into 2 different sets of instructions and were clearly labeled as “green-lidded swab” and “black swab,” with pictures of the swabs.

##### Clarity of Instructions

The participants were not always clear on how to assess features or conduct the tests. The clarity of instructions was improved and we added a video for the swab tests.

##### Suitability of Materials

The participants were unfamiliar with handling medical samples and struggled with the materials that were provided. The method for packing the 2 different swabs required additional explanation and videos. This was complicated by suppliers running low on appropriately sized sample bags during the COVID-19 pandemic. The sample bags we sent out were initially only just big enough and then too large to easily fit in the sample box, so it was important to refine the test kits and match them to the instructions in the final iteration.

### Stage 2—Feasibility Study

#### Participants

**Figure 1:**
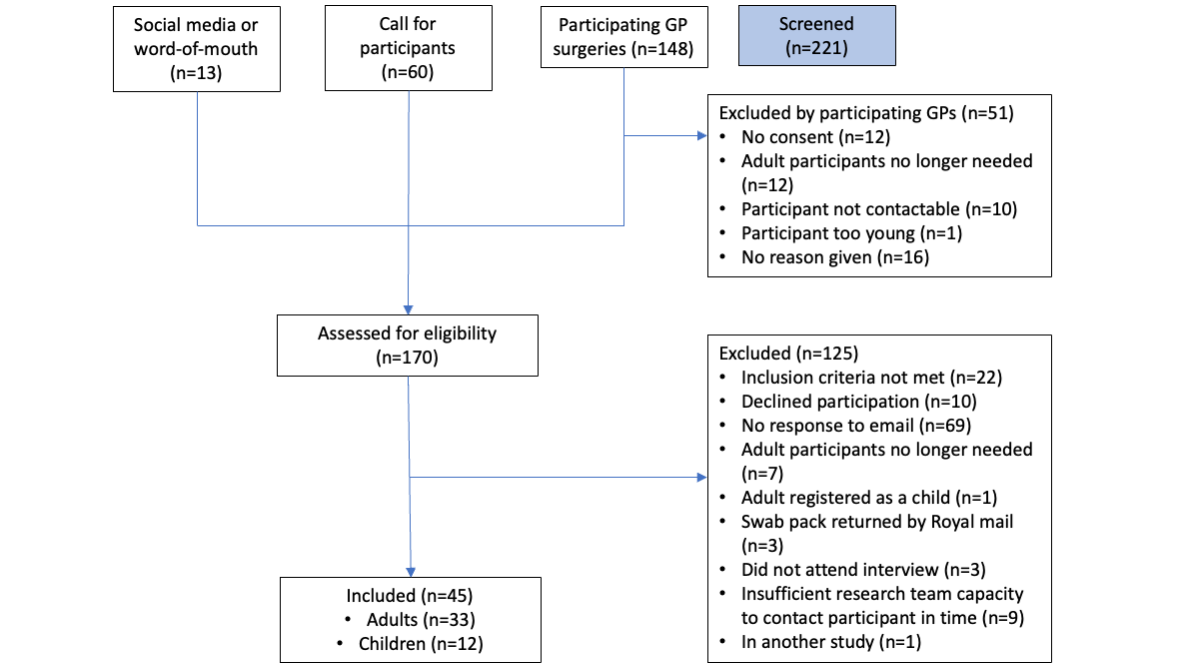
Recruitment flow diagram. GP: general practitioner.

A total of 33 adult and 12 child participants took part in stage 2 (Figure 1). In this stage, 1 (3%) out of 33 adults had missing data on race and ethnicity but there were no other missing demographic data (Table 1). Adult participants were mostly women (23/33, 70%), young (median age 28, IQR 21-38 years), and White (24/32, 75%), with racial and ethnic proportions broadly in line with the UK population (given the small numbers). Child participants were evenly split between genders (6/12, 50% girls), with a median age of 9 (IQR 5.5-12.5) years, and were mostly White (8/12, 67%).

**Table 1:** Characteristics of participants.

Characteristics		Adult participant (n=33)	Child participant (n=12)
Gender (woman or girl), n (%)		23 (70)	6 (50)
Age (years), median (IQR)		28 (21-38)	9 (5.5-12.5)
**Race and ethnicity (adult: n=32; child: n=12), n (%)**			
	Asian	5 (16)	2 (17)
	Black	1 (3)	2 (17)
	White	24 (75)	8 (67)
	Multiracial	1 (3)	0 (0)
	Other	1 (3)	0 (0)
Missing, n (%)		1 (3)	0 (0)

#### Quantitative Findings

##### Self-Assessment and Throat Photos

In this assessment, 1 parent or carer did not complete the questions on pus or painful lymph nodes and a further 18 participants indicated that they were “not sure” for either throat inflammation, painful lymph nodes, or both, meaning that FeverPAIN and Centor scores could be calculated for 35 (78%) and 32 (71%) out of 45 participants, respectively (Table 2).

**Table 2:** Self-assessment of throat.

Variable			Adult participant (n=33)		Child participant (n=12)
Severity score^a^, median (IQR)			6 (4-7)		8 (7-8)
Sore throat started less than 3 days ago, n (%)			10 (30)		3 (25)
Fever, n (%)			7 (21)		4 (33)
Persistent cough, n (%)			3 (9)		3 (25)
Runny or blocked nose or sneeze, n (%)			14 (44)		4 (33)
**Throat very swollen or red, n (%)**					
	Yes	15 (46)		9 (75)	
	No	10 (30)		2 (17)	
	Not sure	8 (24)		1 (8)	
**Yellow or white spots on throat, n (%)**					
	Yes	11 (33)		6 (50)	
	No	22 (67)		5 (42)	
	Missing	0 (0)		1 (8)	
**Painful lymph nodes, n (%)**					
	Yes	14 (42)		5 (42)	
	No	12 (36)		1 (8)	
	Not sure	7 (21)		5 (42)	
	Missing	0 (0)		1 (8)	
**FeverPAIN score, n (%)**					
	0-1	10 (30)		3 (25)	
	2-3	11 (33)		4 (33)	
	4+	4 (12)		3 (25)	
	Missing	8 (24)		2 (17)	
**Centor score, n (%)**					
	0-2	18 (55)		4 (33)	
	3-4	8 (24)		2 (17)	
	Missing	7 (21)		6 (50)	
Received antibiotics, n (%)			7 (21)		7 (58)

^a^Severity score on a scale of 1 to 10.

Most adult participants and parents of child participants were confident that they correctly assessed the throat (9/10, 90% and 26/32, 81%, respectively), did not find it difficult (9/10, 90% and 21/32, 66%), thought the instructions were easy to follow (10/10, 100% and 28/32, 88%), did not find it unpleasant (4/10, 40% and 23/32, 72%), and would be happy to do it again (10/10, 100% and 26/32, 81%; Table 3).

**Table 3:** Participants rating of use of self-assessment tool.

Participant and rating		Confident did test correctly, n (%)	Found it difficult, n (%)	Instructions easy to follow, n (%)	Found it unpleasant or uncomfortable, n (%)	Happy to do it again, n (%)
**Parent of child participant (n=10)**						
	Strongly disagree	0 (0)	4 (40)	0 (0)	1 (10)	0 (0)
	Disagree	1 (10)	5 (50)	0 (0)	3 (30)	0 (0)
	Neutral	0 (0)	0 (0)	0 (0)	4 (40)	0 (0)
	Agree	3 (30)	1 (10)	3 (30)	1 (10)	1 (10)
	Strongly agree	6 (60)	0 (0)	7 (70)	1 (10)	9 (90)
**Adult participant (n=32)**						
	Strongly disagree	0 (0)	12 (38)	1 (3)	12 (38)	0 (0)
	Disagree	1 (3)	9 (28)	1 (3)	11 (34)	1 (3)
	Neutral	5 (16)	5 (16)	2 (6)	4 (13)	5 (16)
	Agree	13 (41)	3 (9)	9 (28)	4 (13)	10 (31)
	Strongly agree	13 (41)	3 (9)	19 (59)	1 (3)	16 (50)

A total of 35 participants (25/33, 76% adults and 10/12, 83% children) provided photographs of their throat. The photographs from 52% (13/25) of adults and 50% (5/10) of children (18/35, 51% of those providing photos and 18/45, 40% of the total study population) were rated as being of sufficient quality for the assessment of the inflammation and pus by 2 or more clinician assessors. The clinician interrater agreement κ score for inflammation was 0.37 (fair) and for pus was 0.42 (moderate). Using clinician assessment as the reference standard, self-assessment of the inflammation and pus was very sensitive but not very specific (Table 4). Overall, there was moderate agreement in the assessment of inflammation (10/18, 56%) and pus (13/17, 76%). Clinician-assessed FeverPAIN and Centor scores were on average lower than self-assessed scores (Table 5).

**Table 4:** Accuracy of self-assessed inflammation and pus as compared to clinician assessment.

Assessment				Clinician assessment, n				Accuracy, n/N (%)		Sensitivity, n/N (%)			Specificity, n/N (%)			PPV^a^, n/N (%)			NPV^b^, n/N (%)	
				Yes		No														
**Inflammation**									56 (10/18)		100 (3/3)			47 (7/15)			27 (3/11)			100 (7/7)
	**Patient or parent assessment**																			
		Yes	3		8		N/A^c^			N/A		N/A			N/A			N/A		
		No	0		7		N/A			N/A		N/A			N/A			N/A		
**Pus**									76 (13/17)		100 (3/3)			71 (10/14)			43 (3/7)			100 (10/10)
	**Patient or parent assessment**																			
		Yes	3		4		N/A			N/A		N/A			N/A			N/A		
		No	0		10		N/A			N/A		N/A			N/A			N/A		

^a^PPV: positive predictive value.

^b^NPV: negative predictive value.

^c^N/A: not applicable.

**Table 5:** FeverPAIN and Centor scores based on self-assessment, clinician-assessment, and Royal College of Paediatrics and Child Health guidance (add 2 to FeverPAIN instead of assessing throat) for participants with an acceptable quality throat photograph of acceptable quality.

Score		Adult participant, n (%)				Child participant, n (%)				Total, n (%)		
		Self-assessed (FeverPAIN: n=12; Centor: n=13)	Clinician assessed (FeverPAIN: n=12; Centor: n=13)	RCPCH^a^ (n=13)	Self-assessed (n=4)		Clinician assessed (n=4)	RCPCH (n=5)	Self-assessed (FeverPAIN: n=16; Centor: n=17)		Clinician assessed (FeverPAIN: n=16; Centor: n=17)	RCPCH (n=18)
**FeverPAIN**												
	0-1	3 (25)	6 (50)	0 (0)	3 (75)		4 (100)	0 (0)	6 (38)		10 (62)	0 (0)
	2-3	7 (58)	5 (42)	9 (69)	1 (25)		0 (0)	4 (80)	8 (50)		5 (31)	13 (72)
	4+	2 (17)	1 (8)	4 (31)	0 (0)		0 (0)	1 (20)	2 (12)		1 (6)	5 (28)
**Centor**												
	0-2	9 (69)	10 (77)	N/A^b^	4 (100)		4 (100)	N/A	13 (76)		14 (82)	N/A
	3-4	4 (31)	3 (23)	N/A	0 (0)		0 (0)	N/A	4 (24)		3 (18)	N/A

^a^RCPCH: Royal College of Paediatrics and Child Health; score based on Royal College of Paediatrics and Child Health guidance (omit the assessment of inflammation and pus and add 2).

^b^N/A: not applicable.

##### Self-Sampling

Analyzable bacterial throat swab samples were received for 89% (40/45) of participants. A total of 4 adult participants did not provide a bacterial swab sample and 1 sample was unlabeled and therefore not processed. Analyzable viral throat swabs were received from 42 (93%) participants. A total of 2 participants (1 adult and 1 child) did not provide viral swab samples and 1 participant (adult) provided an unlabeled sample that was therefore not analyzed. A total of 40 (89%) participants provided analyzable saliva sponge samples. About 2 participants (1 adult and 1 child) did not provide samples and 3 participants (all adults) provided insufficient volume for analysis. A total of 36 (80%) participants provided sufficient saliva drool samples, with 2 (1 adult and 1 child) not providing a sample and 7 (6 adults and 1 child) not providing sufficient volume for analysis.

The saliva pot had the best ratings in terms of how unpleasant the test was to use, with the saliva sponge as slightly more unpleasant and the throat swab as the most difficult and unpleasant (Table 6). However, for all methods, participants generally felt confident they had done it correctly, found the instructions easy to follow, and would be happy to do it again.

**Table 6:** Feasibility of throat self-assessment methods.

Feasibility and participant			Saliva pot (N=45)	Saliva with sponge (adults and children 6+; n=42)		Saliva with sponge (children aged 5 years and younger; n=3)		Throat swabs (n=45)
**I feel confident I did it correctly, median (IQR)^a^**								
	Adult	5 (4-5)		4 (4-5)	N/A^b^		4 (3-4)	
	Child	5 (5-5)		5 (4-5)	4.5 (4-5)		4 (4-5)	
**I found it difficult to do, median (IQR)^a^**								
	Adult	1 (1-2)		2 (1-2)	N/A		3 (2-4)	
	Child	1 (1-1)		2 (1-2)	1.5 (1-2)		4 (1-4)	
**I found the instructions easy to follow, median (IQR)^a^**								
	Adult	5 (4-5)		5 (4-5)	N/A		4 (3-5)	
	Child	5 (5-5)		5 (4-5)	3.5 (3-4)		5 (5-5)	
**I found it unpleasant or uncomfortable, median (IQR)^a^**								
	Adult	1 (1-3)		2 (1-3)	N/A		4 (3-5)	
	Child	1 (1-1)		3 (2-4)	1 (1-1)		4 (3-5)	
**I would be happy to do it again, median (IQR)^a^**								
	Adult	5 (4-5)		5 (4-5)	N/A		4 (3-5)	
	Child	5 (5-5)		5 (4-5)	5 (5-5)		5 (3-5)	
**Number missing, n/N (%)**								
	Adult	4/33 (12)		4/33 (12)	N/A		3/33 (9)	
	Child	1/12 (8)		0/9 (0)	1/3 (33)		1/12 (8)	

^a^On a scale of 1 to 5, where 1=strongly disagree, 2=disagree, 3=neutral, 4=agree, 5=strongly agree.

^b^N/A: not applicable.

#### Qualitative Findings

##### Overview

A total of 29 adults and 9 parents (8 mothers and 1 father) participated in stage 2 interviews. The findings below are based on the following topics.

##### Views About the Self-Assessment Website

Both adult and parent participants were overwhelmingly positive about the design and content of the website, with participants reporting that the website was “easy” to use and the instructions were “clear.”

The questions were really interesting to answer. The instructions were clear, I thought.

Adult participant, 333

Yeah, it was really good. Really easy. The diagrams of the tonsils and stuff was really helpful...I don’t think there’s anything I [would] probably add.

Mother of a child aged 10 years, 217

A few participants suggested minor changes. A total of 3 participants asked for more instructions about how to take a photograph of the throat because they had found this aspect difficult (see the *Taking a Photograph of the Throat* section) and 2 thought that a throat photograph would be better than the diagram provided in helping them identify the different parts of the throat.

##### Self-Assessing a Sore Throat Using Diagnostic Indicators of an Infection

The self-assessment website included instructions on how to self-assess a sore throat using criteria from FeverPAIN and Centor scores. Most adult and parent participants said they found the self-assessment “easy” and the criteria on the website “helpful.”

...when you asked to look at your own throat, how easy did you find that? Were you able to find your tonsils, for example?

Interviewer

Yeah so that was very easy, there were no problems with that.

Adult participant, 317

Yeah, it was easy to follow and the pictures were helpful, um explaining what, what you’re looking for, ‘cause it said you know, sometimes red throats are normal, normal, but it was looking at specific areas of the throat where the redness was, and whether there was pussy bits and things, so it was quite helpful, yeah.

Mother of a child aged 13 years, 215

However, 1 participant described uncertainty when trying to examine for enlarged lymph nodes in the neck:

It was okay. When trying to feel for the neck it was hard to know what’s what, whether, you know, is it the tongue muscle or is it, whatever so. Other than that yeah it was okay.

Adult participant, 316

However, 2 parents indicated that already knew how to self-assess a sore throat because they had done it previously, thus inferring that the website did not provide them with new information about this:

There wasn’t anything [on the website] I would change because those are all the things that I would do normally...So feeling the, feeling his glands and looking at the back of the throat, identifying any swollen areas or red areas or and all the rest of it. So, I mean, that’s, pretty much what we do anyway, isn't it?

Mother of a child aged 12 years, 216

I had to, had to look at her tonsils. She had some, some white spots on it. So she already had tonsillitis last year, so I knew the sign, the symptoms, the signs, yeah.

And how easy was it to look in her mouth and see her tonsils?

Interviewer

Umm it was easy for me.

Mother of a child aged 5 years, 111

##### Taking a Photograph of the Throat

Both adult participants and parents reported difficulties with obtaining, what they perceived to be a “useful” or “clear” photograph of the throat. The challenges primarily related to practical issues such as inadequate lighting, getting the camera to focus inside the mouth, and minimizing the presence of the teeth and tongue in the photograph. Participants reported trying different strategies to improve the quality of the photograph, such as moving rooms and moving the camera further away but also acknowledged that the throat is a dark place and thought it would be difficult regardless.

...What I think I found difficult was the taking of the photograph because however you try to take it seemed, I don’t know whether I was taking enough of my throat or whatever. And obviously, you know it’s not the most attractive photo is it hahaha. But yeah that was the bit because I think however you try to open your mouth just difficult to take that photo yourself.

Adult participant, 320

There’s so many pictures on my phone of just like my tongue and maybe a little bit of my throat, but nothing like—nothing where you could see it.

Adult participant, 351

...but picture thing was an absolute nightmare and obviously again, a bit like what I was saying when she’s at home and there’s not somebody else present she’s not as well behaved. So for me to say to her ‘I need to take a picture of your mouth’ after about the fifth time she was so bored of it that she was just saying, ‘well, no, I’m not opening my mouth anymore.’

OK, did you manage to get a picture?

Interviewer

*I did get the picture. Yeah, but it didn’t show anything other than hertongue and could see her taste buds and that was it.* [Mother of a child aged 5 years, 113]

Participants also expressed negative views about a diagnosis being based on a photograph alone, due to difficulties in obtaining a “clear” image of the throat. Concerns centered on whether the photograph would convey the severity of the infection, possibly resulting in an inaccurate diagnosis and possibly mistreatment, compared to a video consultation or an in-person examination.

I know I can see through mirrors that there is a problem with my tonsils; it’s all red or maybe I have some pus in my tonsils but I couldn’t take a good picture of that.

Adult participant, 339

No. I mean I had two or three goes at it and I don’t think there was a clear view. I think it might have been okay if I had huge tonsils with big white spot on like on the picture but mine was red, a red area. So I don’t think that would be, the quality wasn’t good enough.

Adult participant, 345

I don’t know. I don't think I would have been actually, I don’t think it would have been clear enough for the doctors to see. Funnily enough, when I phoned up the receptionist did ask me to take photo from his throat, but when I spoke to the doctor he didn’t actually ask for that photograph and just from, from my explanation and how he was feeling that, that’s, that’s kind of how he diagnosed it, and so he didn’t even ask for it. So, but I don’t think it would have been clear enough for him to be seen anything, because it was much further back.

Mother of child aged 11 years, 211

##### Self-Sampling

Participants were happy to receive and return the swab and saliva test kits using the postal service and some said that they would also be willing to pick up and complete a kit at their primary care center. Participants generally reported that although the self-sampling tests were not pleasant and sometimes uncomfortable, especially the throat swabs, they were able to complete them. The saliva samples were described as easier to complete than the throat swabs; although some participants reported unanticipated difficulties, such as not being able to produce saliva on demand or enough to fill the line in the sample tube, and difficulty not touching the sample for the test involving the cotton sponge. The main difficulty in completing the throat swabs was that they could make the participant gag, or they could cause pain as the swab had to make contact with the back of the throat which might have still been sore at the time of the interview.

I think the, as I said, the one I found the most difficult was the first one, the spitting the saliva in and, because I didn’t feel I had enough saliva. So because it says you don’t take anything to drink for 15 minutes before, I felt my mouth with a little bit dry, so I had to conjure up the saliva, so that that was the least easy to do.

Participant, 320

Most participants had a preference for self-testing oversampling by a clinician or family member. The reasons given included that they could control the placement of the swab and their gag reflex. Of the participants who said they would have preferred for someone else to assist with the tests, most said that they would have preferred a clinician as they were perceived as being more likely to obtain an accurate sample. A few participants thought that self-testing using the model proposed in this research would be hard for certain groups, such as children, people “without capacity,” and older adults, and said that person-assisted testing would be preferable in these situations.

I was able to do it myself and I was probably more comfortable doing it by myself because I was in control. And you kind of know your own limits with your throat. But for children obviously they would need an adult. But yeah, I thought it was alright.

Participant, 314

Participants mostly reported feeling confident that they had provided a good sample although some were less confident about their throat swabs than the saliva tests. Concerns about the throat swabs related to whether the participant had held the swab in place for long enough and whether they had touched the surrounding area (eg, their tongue or teeth).

Over half of the participants reported having a COVID-19 test before they took part in this study and some of them reported that the experience of self-testing for COVID-19 made testing for the 4S study easier or more familiar.

## Discussion

### Principal Findings

Our study has provided evidence that many patients (and parents of patients) are happy to assess sore throats using FeverPAIN and Centor criteria with the help of a carefully developed website. However, further work is required to determine the clinical use of self-assessment of sore throat. As expected, participants found it more difficult to assess clinical signs, such as swollen lymph nodes, throat pus, and inflammation, than symptoms. The majority of participants were able to send photographs of their throats, but only 51% (18/35) of these were judged to be of sufficient quality to assess inflammation and pus. Participant ratings had high sensitivity and NPVs for both inflammation and pus. However, the specificity and PPVs were much lower (specificity: 10/14, 71% and PPV: 3/7, 43% for pus and specificity: 7/15,47% and PPV: 3/11, 27% for inflammation) suggesting that patients were much more likely to rate themselves as having moderate or severe inflammation and pus than clinicians. Qualitative findings were consistent with the quantitative findings, indicating that most participants thought that the self-assessment tool was useful and that they were able to assess most aspects. Views about self-testing with throat swabs and saliva samples were also broadly positive although some participants were uncertain about whether they had been able to provide a good throat swab sample. Concerns were also expressed about self-photography of the throat, with many participants indicating that they were unable to obtain a good picture. This was also consistent with the quantitative data that found that only 40% (18/45) of the study population provided a photograph of sufficient quality to assess inflammation and pus.

### Strengths and Limitations

Strengths of this study include the development of the intervention using principles of the PBA, the development and testing of the intervention in different populations, and the use of quantitative and qualitative approaches to assess its use in adults and children with sore throat. Limitations include the small sample size and the low proportion of patients able to provide a throat photograph of adequate quality. Allowing participants with sore throats lasting up to 14 days may have resulted in the overrepresentation of those with more prolonged symptoms. Some participants had already seen a primary care clinician, and this may have influenced their assessment of their features; however, this was not mentioned by any participants. In addition, the study was conducted during the COVID-19 pandemic and a throat examination by a health care professional was discouraged at that time. Therefore, the study was conducted entirely remotely and we were not able to obtain in-person clinical assessments of throat inflammation and pus, to compare with patient assessments. As a result, we had to use clinician assessment of the throat photographs taken by participants, and many participants were not able to provide good-quality photographs. Nevertheless, we only used photographs assessed as being of sufficient quality and all photographs were assessed independently by 3 clinicians, with reasonable interrater agreement. Finally, the qualitative interviews did not sufficiently probe participants’ understanding of the different parts of the throat or clinical indicators of severity.

### Comparison With Prior Work

Previous studies have also found differences between patient and clinician assessment of clinical signs related to sore throat. In 1 study, participants were asked to self-assess Centor criteria guided by color images and found that patients and parents were less likely to report tender lymph nodes than clinicians (13% vs 27%) but more likely to report tonsillar exudate (25% vs 19%) [17], which is in keeping with our findings. Another study provided 200 adults with a simplified drawing of the oral pharynx anatomy, flashlights, and mirrors and asked them to record their perception of redness, the presence of the white spots, and whether the tonsils appeared swollen. They found that agreement between clinician and patient assessment of tonsillar exudate was higher than that for pharyngeal erythema [18]. It is therefore likely that self-assessment could lead to overtreatment without further testing or clinician assessment.

### Implications

Our data provide evidence supporting the feasibility of self-assessment for sore throats in the community guided by a website developed with patients and for self-testing with swabs and saliva samples. However, we found that many patients had difficulty taking photographs of their throat and only 40% (18/45) were able to provide photographs of sufficient quality to allow assessment of inflammation and pus. Our comparison between self-assessment of inflammation and pus, and an assessment by the clinicians of the photographs that were of sufficient quality, suggests that patients may overrate pus and inflammation. In our study, self-assessed FeverPAIN scores would have resulted in a recommendation for delayed antibiotics in 50% (8/16) of cases and immediate antibiotics in 12% (2/16) of cases compared with 31% (5/16) and 6% (1/16) for clinician-assessed scores (using photographs), respectively. Using guidance from the RCPCH, which recommends adding a score of 2 in lieu of assessing throat inflammation and pus, would have resulted in 72% (13/18) and 28% (5/18) being prescribed delayed and immediate antibiotics, respectively. Further work is needed to determine if it is valid, safe, and acceptable to use self-reported signs and symptoms to identify patients at low and high-risk of having GABHS, in order to better guide antibiotic therapy. This strategy has the potential to reduce both clinician workload and inappropriate antibiotic prescribing. Low-risk patients could potentially be guided to self-management and safety-netting advice and high-risk patients could be further assessed by clinicians and considered for immediate or delayed antibiotics. Antibiotic use should be low in the intermediate group as long as very clear guidance is given to patients using FeverPAIN about when to use a delayed prescription [20]. It may also be possible to further stratify intermediate patients using testing from either swabs or saliva although further work is needed to determine this [10]. Self-assessment and self-sampling, if shown to be valid are reliable, could also be used to increase the efficiency of recruiting participants into research studies on sore throat.

### Conclusions

We have developed a web-based tool that adult patients and parents or carers of child patients with sore throats found useful and acceptable in helping them self-assess clinical scoring criteria and obtain self-sampled throat swabs and saliva samples. Less than half the participants were able to provide photographs of acceptable quality and so additional support may be required for this aspect. Agreement between patient- and clinician-assessed clinical criteria was moderate, with patients tending to rate clinical features as present more often than clinicians, and subsequently to have higher FeverPAIN and Centor scores. We have demonstrated that the use of this tool is acceptable and feasible, and it now requires evaluation before implementation in clinical practice.

## Appendix

Multimedia Appendix 1 Process evaluation interview topic guide.

## Abbreviations

GABHS: group A β-hemolytic streptococcus
NHS: National Health Service
NPV: negative predictive value
PBA: Person-Based Approach
PPV: positive predictive value
RCPCH: Royal College of Paediatrics and Child Health
4S: Scores and Swabs to Self-Assess Sore Throat

## Data Availability

The data sets generated and analyzed during this study are available from the corresponding author on reasonable request.
