# Correction: Internet Tool to Support Self-Assessment and Self-Swabbing of Sore Throat: Development and Feasibility Study

**DOI:** 10.2196/59420

**Published:** 2024-04-26

**Authors:** Mark Lown, Kirsten Smith, Ingrid Muller, Catherine Woods, Emma Maund, Kirsty Rogers, Taeko Becque, Gail Hayward, Michael Moore, Paul Little, Margaret Glogowska, Alastair Hay, Beth Stuart, Efi Mantzourani, Christopher R Wilcox, Natalie Thompson, Nick A Francis

**Affiliations:** 1 Primary Care Research Centre, University of Southampton Southampton United Kingdom; 2 School of Computing, University of Portsmouth Portsmouth United Kingdom; 3 School of Healthcare Enterprise and Innovation, University of Southampton Southampton United Kingdom; 4 Local Clinical Research Network Wessex Southampton United Kingdom; 5 Nuffield Department of Primary Health Care Sciences, University of Oxford Oxford United Kingdom; 6 Centre for Academic Primary Care, Bristol Medical School, University of Bristol Bristol United Kingdom; 7 Pragmatic Clinical Trials Unit, Queen Mary University of London London United Kingdom; 8 Cardiff School of Pharmacy, Cardiff University Cardiff United Kingdom

In “Internet Tool to Support Self-Assessment and Self-Swabbing of Sore Throat: Development and Feasibility Study” (J Med Internet Res 2023;25:e39791) the authors noted one error.

In the originally published article, the affiliations appeared as follows:

Mark Lown1 , PhD; Kirsten A Smith1 , PhD; Ingrid Muller1 , PhD; Catherine Woods1 , PhD; Emma Maund2 , PhD; Kirsty Rogers3 ; Taeko Becque1 , PhD; Gail Hayward4 , DPhil; Michael Moore1 , BMBS; Paul Little1 , MD, MRCP, MRCGP; Margaret Glogowska4 , PhD; Alastair Hay5 , MD; Beth Stuart6 , PhD; Efi Mantzourani7 , MPharm, MSc, PhD; Christopher R Wilcox1 , MRCGP; Natalie Thompson1 , MSc; Nick A Francis1 , BA, MD, PhD

*1. School of Computing, University of Portsmouth, Portsmouth, United Kingdom*

*2. School of Healthcare Enterprise and Innovation, University of Southampton, Southampton, United Kingdom*

*3. Local Clinical Research Network Wessex, Southampton, United Kingdom*

*4. Nuffield Department of Primary Health Care Sciences, University of Oxford, Oxford, United Kingdom*

*5. Centre for Academic Primary Care, Bristol Medical School, University of Bristol, Bristol, United Kingdom*

*6. Pragmatic Clinical Trials Unit, Queen Mary University of London, London, United Kingdom*

*7. Cardiff School of Pharmacy, Cardiff University, Cardiff, United Kingdom*

The affiliations have been corrected as follows:

Mark Lown1 , PhD; Kirsten Smith2 , PhD; Ingrid Muller1 , PhD; Catherine Woods1 , PhD; Emma Maund3 , PhD; Kirsty Rogers4 ; Taeko Becque1 , PhD; Gail Hayward5 , DPhil; Michael Moore1 , BMBS; Paul Little1 , MSc, PhD; Margaret Glogowska5 , PhD; Alastair Hay6 , MD; Beth Stuart7 , PhD; Efi Mantzourani8 , MPharm, MSc, PhD; Christopher R Wilcox1 , MRCGP; Natalie Thompson1 , MSc; Natalie Thompson1 , MSc; Nick A Francis1 , BA, MD, PhD

*1. Primary Care Research Centre, University of Southampton, Southampton, United Kingdom*

*2. School of Computing, University of Portsmouth, Portsmouth, United Kingdom*

*3. School of Healthcare Enterprise and Innovation, University of Southampton, Southampton, United Kingdom*

*4. Local Clinical Research Network Wessex, Southampton, United Kingdom*

*5. Nuffield Department of Primary Health Care Sciences, University of Oxford, Oxford, United Kingdom*

*6. Centre for Academic Primary Care, Bristol Medical School, University of Bristol, Bristol, United Kingdom*

*7. Pragmatic Clinical Trials Unit, Queen Mary University of London, London, United Kingdom*

*8. Cardiff School of Pharmacy, Cardiff University, Cardiff, United Kingdom*

The correction will appear in the online version of the paper on the JMIR Publications website on April 26, 2024, together with the publication of this correction notice. Because this was made after submission to PubMed, PubMed Central, and other full-text repositories, the corrected article has also been resubmitted to those repositories.

