# Supplementary material for: Internet Tool to Support Self-Assessment and Self-Swabbing of Sore Throat: Development and Feasibility Study
Source: J Med Internet Res. 2023 Dec 8;25:e39791. doi: 10.2196/39791 (PMC10746968; doi:10.2196/39791)
Supplement: Multimedia Appendix 1 [file jmir_v25i1e39791_app1.pdf]

4S Study - Process Evaluation Topic Guide - Semi-structured interview schedule – Stage 2

NOTE: Some kits have different boxes and bags – offer guidance on packing these.

Introductory script (you can vary slightly from this)

I'm XXXX, a researcher at the university of Southampton. First of all, thank-you for agreeing to take part. This study aims to find a good way of testing sore throats at home, without needing to see a doctor. To do this, we are trying out a few different throat tests and self-reported symptoms.

This interview has (2) 3 parts:

- *First of all, you will look at the website.*
- *Then you can try out the test kits.*
- **ONLY if they consented to point 9 (consent to be interviewed):** *Then I'll ask you a few questions about your experience.*

I will be watching you on the webcam to see how you get on – I won't be speaking or asking questions very much until you are finished with the kits.

When we get started, please let me know if you want to take a break for any reason. We can stop there, arrange to start a little later or arrange another time to do the interview.

If a child aged over 11 is present:

If it's okay with you, I would like to check with your child to see if they are happy to take part.

Take Assent from child

Do you have any questions before we start?

Answer questions

Please open the website we sent you. Your code is XXX. Let me know when you are on it, and I will start the recording.

Answer any queries during the interview by encouraging them to work it out for themselves. E.g.

- *'What do you think you should do?'*

- *'Tell me what is difficult.'*
- *'What is unclear?'*
- *'What would you do if I weren't here?'*

Make a note if they do something wrong, and provide instruction if they get frustrated.

Interview questions ONLY if they consented to point 9

**Section 1: General experiences of the advice / information**
Q1. I'm really interested in hearing your views on the information and advice provided, can you tell me what you thought about it?
Q2. Can you tell me about anything you liked about the information and advice? *[Prompts: Why? Anything that made it easy to follow the advice?]*
Q3. Can you tell me about anything you disliked about the information and advice? *[Prompts: Why? Anything that made it difficult for you to follow the advice?]*
Q4. How would you feel about using a tool like this by yourself without a doctor?
Q5. *Do you think this website was missing anything that you expected to see?*
Clinical scores / assessment – this was completed on the consent website
Q6. Can you tell me about how you found assessing [your / your child's] sore throat? Ask about individual parts e.g. pus on tonsils, temperature. Were there any problems? Do you think anything could be improved? Is there anything you would change?
Q7. How did you find taking a photograph of the throat? Would you be happy to send this to a doctor to look at?
Conducting tests
Q8. Can you tell me about how you found taking a swab of [your / your child's] sore throat? Were there any problems? Do you think anything could be improved? Is there anything you would change?
Q9. Have you had a COVID test before? If yes, how has this experience helped (or not helped) you today?
Q10. For participants who have not had a COVID test – How has your experience today affected any future COVID test you might need?
Q11. Can you tell me about how you found taking the saliva samples? Were there any problems? Do you think anything could be improved? Is there anything you would change?

Q12. What do you think about the way the information was presented? What did you think about the format (written / video etc)? How did you find using the website? What did you think of the videos?<br><br>Q13. Did you require someone else to help with any of the procedures or use any tools such as a mirror?<br><br>Q14. Do you feel comfortable and confident in the assessments and tests you did today?<br><br>*Parents and carers only:*<br>Do you feel comfortable and confident in the assessments and tests for your child? Would you be prepared to do it again?<br><br>*Time period*<br><br>Q15. How long did you wait between hearing about this study and receiving your kit? How long would you be happy to wait?<br><br>Q16. How long would you expect to wait to receive results and be offered treatment, after sending the kit back?<br><br>Q17. Would you be willing to wait longer if you knew the treatment would be more likely to work?<br><br>Q18. Would you prefer to post your sample, or pick up a kit at your GP surgery?<br><br>Q19. How many days would you wait from having a sore throat to contacting your GP? Would this have been different pre-covid? In what ways do you think covid has changed seeing your GP for a sore throat?
**Section 2: Finish**
Q20. Do you have anything else you would like to tell me about your experiences of the advice and trial procedures that we haven't already covered?

End of interview

Thank you so much, you've been really helpful. I will stop the recording now.

Stop recording

There are a few final things I would like to mention:

- Is there anything you would like to ask me about the study?
- Can you tell me where you found out about the study?
- Would you like us to send you a copy of the results by email? This will be available in a few months' time.

- **IF they haven't sent a photo:** I'll resend the email asking for the photo of your throat. It would be great if you could reply to that as soon as you can.
- Please put those boxes in the postbox today if you can.
- I will send you the link to your shopping voucher through email within the next week. It is a 'Love to Shop' voucher that you can spend at many different high street stores and online.

Great, that's everything. It's been a pleasure to speak to you, thank-you for your time!
